# The Mediating Role of Self-Concept between Sports-Related Physical Activity and Mathematical Achievement in Fourth Graders

**DOI:** 10.3390/ijerph16152658

**Published:** 2019-07-25

**Authors:** Laura C. Dapp, Claudia M. Roebers

**Affiliations:** Department of Psychology, University of Bern, Hochschulzentrum vonRoll, Fabrikstrasse 8, 3012 Bern, Switzerland

**Keywords:** physical activity, sports experience, exercise, self-concept, mathematical achievement, children, serial mediation

## Abstract

Being physically active has many benefits for children and adolescents. It is essential for various aspects of physical and mental health, and also positively influences academic performance and school achievement. In addressing the still incomplete understanding of the link between physical activity (PA) and academic achievement, the present study scrutinized the open issues regarding different roles of PA type and PA duration within the relation between self-reported PA and objectively measured mathematical achievement in fourth graders. As to the type of PA, the current study distinguished between structured and unstructured sports activities children perform in their leisure time. Moreover, the current study investigated the indirect effect of PA on mathematical achievement by controlling for the mathematical self-concept as a mediating variable. Results showed PA to be positively related to mathematical outcomes if implemented in a structured setting and pursued for at least two hours per week. As to the mediation, the results revealed a full mediation, indicating PA to improve mathematical self-concept, which, in turn, positively affected mathematical achievement. Thus, engaging in structured PA for two hours or more a week may have additional benefits for children’s and adolescents’ self-concept in a way that is favorable and encouraging for promoting their academic achievement.

## 1. Introduction

Being physically active has many benefits, not only for adults but also for children and adolescents. Physical activity (PA) is essential for various aspects of children’s physical and mental health, it contributes to psychological well-being, and it promotes the development of a positive self-concept [1,2,3,4,5]. Moreover, PA plays a vital role in physical, social, and emotional development, since it offers ample opportunities for acquiring and practicing new skills in many different domains of children’s lives [3,6].

A growing body of literature suggests that PA may also have beneficial effects on children’s cognitive outcomes. For example, PA has been shown to have a positive impact on children’s cognitive performance, academic behavior, and precursors of academic achievement, such as concentration, time on task, and classroom behavior [7,8,9,10]. Moreover, PA has been found to benefit academic achievement in different academic domains, such as reading, spelling, or mathematics [11]. Although a substantial and direct link between PA and academic achievement could not be confirmed unequivocally by given research [7], meta-analyses report significant and positive effects of PA on children’s cognitive performance with small to moderate effect sizes [12,13]. Moreover, reviews consistently conclude that increasing the amount of PA—even if on behalf of other subjects—either results in improvements in children’s academic achievement or remains without any effect. However, increased PA has not been found to adversely affect school performance in the academic subjects [7,8,13].

Despite numerous studies documenting a positive relationship between PA and children’s academic achievement, the overall inconsistent findings have led to a debate whether or not PA truly and directly improves children’s academic achievement [12]. The inconsistency of the findings may be explained by the reliance on different conceptual definitions of PA and cognitive outcomes, as well as by the non-consideration of potential mediators. Regarding the characteristics of academic outcomes, it has been suggested that particularly mathematical skills may be susceptible to being enhanced by PA [11,12]. As to the operationalization of PA, it has to be noted that the reliance on the various subcategories of PA may have revealed different findings regarding cognitive outcomes. In the broadest sense, PA refers to any bodily movement that is produced by skeletal muscles and results in energy expenditure [14]. Thus, by definition, PA includes any movement that occurs while sleeping, working, or during leisure time. The latter provides children with the opportunity to engage in physically active play, sports, and exercise, that is, various forms of more intense bodily movement. Sports-related leisure-time PA (hereafter simply referred to as “PA”) may thus—in addition to school-based physical education—be the most relevant subcategory of PA when it comes to foster cognitive outcomes in primary school children. However, it is still unclear how different (sub)types of PA may affect cognitive outcomes. Similarly, it is unknown for how long one needs to engage in PA to improve his or her academic achievement [15,16]. Regarding the mediators, it has been assumed that the link between PA and academic outcomes may be rather indirect, that is, that a third construct might underlie this association. In addressing these unanswered questions, the present study sought to explore the aspects of PA type, PA duration, and mediators in more detail.

### 1.1. Type of Physical Activity

PA can be classified in many different ways. One way to distinguish different types of PA is by its level of organization and structuredness [17]. Accordingly, PA can be classified into structured (formal) exercise and deliberate sports practice (hereafter simply referred to as “structured PA”) and unstructured (non-formal) physically active play and sports activities (hereafter simply referred to as “unstructured PA”). Structured PA is thereby defined as adult-led sports activities, including all kinds of organized training, and aims to improve participants’ performance. Structured PA is always guided by a trainer, teacher, or other sports authorities and takes place regularly and at fixed times [14,18]. In contrast, unstructured PA describes spontaneously created, peer-led physical activities that are performed in playful environments [19]. These may also be related to structured PA in some cases (e.g., children being in a soccer club kicking balls after school, though neither planned nor guided by a sports authority), but refer to all forms of informal sports-related activities performed by children on their own in their leisure time [18,19].

The majority of the studies addressing the relationship between PA and children’s academic outcomes examined the effects of sports-related PA as provided by school faculty, i.e., school-based PA [12,20]. By definition, school-based PA includes curricular physical education, classroom-based PA, integrated PA, extracurricular PA, and active recess [7,11]. The major part of school-based PA is structured in its nature because it is mostly guided by teachers. In this context, researchers have suggested that, given the proximity of the educational environment, (structured) PA is more likely to benefit academic outcomes if offered at school rather than in other contexts [8]. They argue that engaging in school-based PA may positively affect the relationship between pupils and teachers, strengthen the sense of belonging to one’s school, and foster the social and emotional connection to school; hence, outcomes increasing the motivation and investment in school [6,8,11,21]. While numerous studies have supported the assumption that engaging in school-based PA has positive effects on children’s academic achievement, little is known about the effects of structured PA realized in a setting that has no reference to school.

### 1.2. Duration of Physical Activity

Another pending issue concerns the quantity of PA needed to positively affect children’s academic achievement, in other words, the search for the optimal amount of PA when it comes to benefiting cognitive outcomes [16,22]. While a minimal amount of PA seems to be needed to foster children’s academic achievement at all, it is yet unclear what the optimal duration of PA for cognitive benefits is [23]. Researchers have suggested that increased amounts of PA might be positively associated with an improvement in academic achievement, but only so to an upper, however unknown, margin. Hence, further increasing the amount of PA might not yield any further improvements. On the contrary, spending too much time in PA might yield negative effects on achievement, as it could distract children from pursuing their school-related goals [24].

### 1.3. Self-Concept as a Mediating Variable

Ultimately, the mechanisms behind the positive effects of PA on academic achievement are still far from being fully understood [15]. PA is assumed to enhance cognition and academic performance via multiple pathways, including physiological and psychological factors [15,16]. Given the predominance of brain-related approaches, where improvements in academic achievement are explained by changes in brain function and brain structure due to PA [15,25], the present study’s focus is directed at a psychological factor, namely the children’s self-concept.

It has been put forward that PA might increase the self-concept, which, in turn, could have positive effects on children’s motivation and academic achievement [26,27]. In an early theoretical work, Snyder had already discussed the possibility that PA might enhance academic performance because experiences of success in sports might lead to a heightened sense of self-worth that spills over into academic achievement [26]. On a theoretical level, this assumption is conceptualized in Sonstroem’s exercise and self-esteem model [28], which provides a rationale for general self-esteem enhancement through sports participation. Simply put, the exercise and self-esteem model suggests that engaging in PA increases the physical self-concept, which, in turn, results in an increased general self-concept. From a top-down perspective, then, a higher general self-concept is also associated with a higher academic self-concept, which finally positively affects academic achievement [29].

However, and even though there is broad agreement about the assumption of self-concept being a critical mediating variable within the nexus of PA and academic achievement, only a few studies have explicitly investigated a mediation of PA on academic achievement by self-concept [27]. The reason for the lack of such studies may lie in the dominance of domain-specific associations between self-concept and objective measures. Indeed, studies investigating the relationship between PA and self-concept clearly show the positive effects of PA on children’s self-concept [2,30,31]. Similarly, self-concept has repeatedly been shown to be directly related to academic achievement [32,33]. However, these associations are predominantly domain-specific. That is, the strongest effects of PA on self-concept are found for the physical self-concept, whereas academic outcomes are predominantly related to the academic self-concept. Thus, while—within a multidimensional self-concept [34]—mathematical self-concept is seen as the strongest correlate for mathematical achievement [35,36], only a little research has been conducted to provide evidence that PA may be related to mathematical self-concept. Nevertheless, the few studies that investigated the cross-domain effects of PA on self-concept suggest academic self-concept to be a potentially crucial mediating variable when considering the effects of PA on a range of academic outcomes [27,37].

### 1.4. The Present Study

The present study investigated the effects of structured and unstructured PA executed in school-unrelated settings on mathematical achievement. In Switzerland, a minimum of three lessons of curricular physical education per week is compulsory in primary schools. For the present study, this characteristic constitutes an advantage since all children receive an equal and constant amount of (structured and school-based) PA. At the same time, extracurricular after-school PA programs or school sports are rather unusual in Switzerland. In contrast, children have the possibilities to engage either in sports-like activities created by themselves (unstructured PA) or in sports provided by a non-school-based sports club (structured PA). Taken together, these circumstances allow for considering the effect of structured PA on academic achievement purified from the assumed strengthening effect on children’s sense of belonging to their school.

In addressing the duration-response relationship, we compared children with low temporal PA engagements to children with high temporal PA engagements regarding their mathematical achievements and self-concepts. Since measures of PA, mathematical self-concept, as well as mathematical achievement, are known to be sensitive to gender, family background, and body composition [24,38,39,40], corresponding control variables were taken into account. Finally, the assumed self-concept enhancement through PA, which, in turn, should affect mathematical achievement, was investigated by a mediation analysis. Hence, the present study addressed three widely open standing issues within the research area of PA and academic achievement in children, namely the influence of PA type and PA duration, as well as a mediating role of self-concept.

## 2. Materials and Methods

### 2.1. Procedure

Trained experimenters assessed PA, self-concept, and mathematical achievement in fourth-grade children. The assessment took place in a classroom setting. All questionnaires were in paper-and-pencil format and put together to a little booklet that was distributed to the children as they were sitting at their desks. The order of the questionnaires was fixed, starting with the assessment of background variables and followed by the questionnaires evaluating PA, then self-concept, and, finally, mathematical achievement. At any given time, all children were working on the same type of questionnaire. Quicker children were told to wait silently until their classmates completed the task and were not allowed to move to the next page until the subsequent questionnaire was instructed by the experimenter. The entire test session took about 45 min.

Schools were enquired randomly for participation. Only classes with given informed consent by the headmaster as well as a class teacher were tested. This resulted in ten classes from eight different schools. The study had been approved by the Faculty of Humanities’ Ethics Committee (Nr. 2017-10-00003) at the University of Bern, Switzerland, and was realized per the Declaration of Helsinki. Parents gave written informed consent for their children to participate in the study, and children orally agreed to participate before the testing started.

### 2.2. Participants

One hundred and forty six children (71 girls, 75 boys) from the German-speaking part of Switzerland participated in the study. Five children were absent from assessment. Three children were known to be training in squads, and since their weekly training duration exceeded the value of the third median absolute deviation (MAD) [41], they were excluded from analyses. Thus, the final sample consisted of *N* = 138 (48.6% girls). A power analysis using the Gpower program [42] revealed 95% power to detect moderate correlations [43] and 83% power to detect moderate effect sizes in comparing two groups with *N* = 138 and alpha at the 0.05 level (two-tailed). At the time of data collection, children attended fourth grade with a mean age of 10 years and 4 months (*SD* = 5.74 months).

### 2.3. Materials

#### 2.3.1. Mathematical Achievement

The mathematical achievement was assessed by three subtests of the “Heidelberger Rechentest” (HRT 1-4) [44], a standardized and curriculum-based test of mathematical achievement for German-speaking primary-school children. The used subtests were: Addition, subtraction, and multiplication. The reported test-retest reliabilities (*r_tt_*) of the HRT subscales are *r_tt_* = 0.82 for addition, *r_tt_* = 0.86 for subtraction, and *r_tt_* = 0.80 for multiplication. Regarding the validity of the HRT, a correlation of *r* = 0.67 with the achieved grade in mathematics has been reported [44]. For each subtest, children first solved three samples to ensure that they understood the (arithmetical) task. For each subtest, children had two minutes to solve as many mathematical problems as possible in sequence. Scores were computed according to the number of correct answers unless more than two problems in the sequence were omitted. Each subtest consisted of 40 tasks. No child attained the maximum score. The tests’ intercorrelations were all significant at *p* < 0.001, and the internal consistency across all three subtests was a Cronbach’s α of 0.87. Thus, the average of correctly solved tasks across the three subtests was used as the dependent variable.

#### 2.3.2. Physical Activity

In the current study, two types of sports-related leisure-time PA were investigated. Structured PA was defined as all kinds of sports performed within so-called sports clubs under the guidance of a trainer (e.g., soccer club, gymnastics club, track-and-field club). Unstructured PA, in contrast, was defined as all kinds of sports-like activities performed by children on their own or among peers (e.g., playing basketball after school, inline skating in the neighborhood).

Structured and unstructured PA were assessed by a sub-questionnaire of the Motorik Modul (MoMo) [45]. The overall reported test-retest reliability of the MoMo is *r_tt_* = 0.83 [46]. More specifically, reliabilities of k = 0.87 (*SD* = 0.12) have been reported for structured PA and k = 0.78 (*SD* = 0.13) for unstructured PA [46]. As to the validity of the MoMo, both structured (*r* = 0.56, *p* < 0.05) as well as unstructured PA (*r* = 0.65, *p* < 0.05) have been shown to be significantly related to activity measures obtained by accelerometers [46].

For structured PA, children had to state whether they were a member of one or more sports clubs or not at all, and, if ever they were, to note all types of sports they were practicing in sports clubs. For unstructured PA, children had to list all sports activities they were performing outside of a sports club. For each pursued sport, either structured or unstructured, children had to specify the weekly frequency of training as well as the average duration of a training session. This allowed for computing the average duration (in hours per week) a child spent in structured or unstructured PA, respectively.

For the unstructured PA, the third MAD was computed similar to the structured PA third MAD (see above). However, exceeding values were adjusted to the third MAD, rather than excluded from the analyses.

For the analyses reported below, PA participation was organized into a dichotomous variable (participation versus non-participation). Mean duration of weekly performed PA in hours was implemented as a continuous variable. In addressing the duration-response issue, children engaging in structured PA were categorized into groups of children spending up to two hours per week (low-duration) and children spending two hours or more per week (high-duration) in structured PA.

#### 2.3.3. Self-Concept

Mathematical self-concept was evaluated by the respective subscale of the Self-Description Questionnaire (SDQ-I) [47,48]. The reported test-retest reliabilities for the mathematical subscale of the SDQ-I are between *r_tt_* = 0.61 and 0.68 [49]. As to the validity of the SDQ-I, correlations up to *r* = 0.36 have been reported with the achieved grade in mathematics [36]. Since the internal consistency across the eight items assessing mathematical self-concept was high (Cronbach’s α = 0.95), a mean score for mathematical self-concept was computed.

#### 2.3.4. Control Variables

In controlling for potential confounding factors, participants’ age, gender, family background in terms of socioeconomic status (SES), and body composition in terms of body mass index (BMI) were taken into account. To compute BMI (kg/m^2^), the weight (kg) and height (m) of every child were measured. SES was evaluated by seven items of the Family Affluence Scale (FAS III), a questionnaire assessing the material resources, patterns of consumption, and purchasing power of a child’s family [50].

### 2.4. Analysis Plan

The data set comprised four items with missing values, all of them concerning the assessment of mathematical self-concept. A Little’s test of missing completely at random (MCAR) for all variables of interest was insignificant, *χ*^2^ (48) = 52.65, *p* = 0.30, revealing that the missing data was completely at random [51]. Since the proportion of missing values was very small (less than 1%), a single imputation procedure was eligible to provide unbiased parameter estimates, such that bootstrapping for estimating indirect effects and improving statistical power was manageable [52]. Thus, a Maximum Likelihood single imputation procedure using the Expectation-Maximization (EM) algorithm in SPSS 25.0 (IBM Corp., Armonk, NY, USA) was performed [52].

Tests of normality showed the weekly duration of structured and unstructured PA, as well as the mathematical self-concept to be non-normally distributed (QQ-Plots; Shapiro-Wilk tests, *p* < 0.001). Consequently, nonparametric tests were applied for non-normally distributed variables (i.e., Spearman rank-order correlations (*r_s_*), Mann-Whitney-U-Tests, Wilcoxon-Signed-Rank-Tests).

To investigate the hypothesis that mathematical self-concept mediated the effect of PA on mathematical achievement, Maximum Likelihood mediation analysis was applied in Amos 25.0 software [53]. To test the significance of direct and indirect effects, a 2000 resampling bootstrapping procedure for the 95% bias-corrected confidence intervals was executed [54,55].

## 3. Results

### 3.1. Overview

The descriptive statistics are presented first, followed by a description of the link between mathematical achievement and structured and unstructured PA, respectively. As there was a substantial correlation between structured PA and mathematical achievement, this relationship was elaborated on by comparing the low-duration with the high-duration children. Finally, the indirect effect of structured PA on mathematical achievement, as mediated by mathematical self-concept, is presented.

### 3.2. Preliminary Analysis

Preliminary analyses revealed the variables of interest to be unaffected by confounding factors. BMI was uncorrelated with mathematical self-concept (*r* = −0.04), mathematical achievement (*r* = −0.16), time spent in structured PA (*r* = −0.13), and time spent in unstructured PA (*r* = −0.10). SES was uncorrelated with mathematical self-concept (*r* = 0.08), mathematical achievement (*r* = −0.06), and time spent in structured PA (*r* = 0.17). The only significant correlation was found between SES and time spent in unstructured PA (*r* = 0.26, *p* < 0.05), wherefore we controlled in the following analyses. Participants’ age was uncorrelated with mathematical self-concept (*r* = 0.07), mathematical achievement (*r* = −0.04), time spent in structured PA (*r* = −0.02), and time spent in unstructured PA (*r* = −0.07). As to the participants’ gender, significant differences were found regarding mathematical self-concept and time spent in structured and unstructured PA. Detailed results on gender differences are reported in the descriptive statistics.

### 3.3. Descriptive Statistics

#### 3.3.1. Mathematical Achievement and Mathematical Self-Concept

Means and standard deviations of analyzed variables are shown in Table 1. Children scored in ordinary test ranges on both mathematical achievement and mathematical self-concept. The mathematical achievement ranged from 11.67 to 33.67, whereas the mathematical self-concept from one to five. No significant gender difference could be discerned for mathematical performance, *t*(126) = 1.67, *p* = 0.10; however, a Mann-Whitney-U-Test revealed the boys had a significantly higher mathematical self-concept compared to girls, Z = −3.62, *p* < 0.001.

#### 3.3.2. Physical Activity Participation

Overall, 91.3% of all children reported participating in PA. With regard to structured PA, 74.6% of the children (68.7% of the girls; 80.3% of the boys) reported to be a member of at least one sports club; 20.3% of the children (23.9% of the girls; 16.9% of the boys) were in more than one sports club. Among the 25.4% of the children who did not participate in structured PA, 10.9% had been sports clubs members in the past but quit their commitment.

With regard to the unstructured PA, 55.1% of the children (46.3% of the girls; 63.4% of the boys) reported to regularly engage in at least one type of PA outside a sports club, and 29.0% (22.4% of the girls; 35.2% of the boys) indicated to pursue more than one type of unstructured PA.

Finally, 38.4% of the children (26.9% of the girls; 49.3% of the boys) reported to engage in both structured and unstructured PA, and 8.7% of the children (11.9% of the girls; 5.6% of the boys) reported to engage in neither structured nor unstructured PA.

Chi-square tests of independence were performed to examine the relationship between gender and frequency of PA participation. The results for structured PA indicated that the girls’ and boys’ respective behavior did not differ, *χ*^2^ (1, *N* = 138) = 2.46, *p* = 0.12 (Cramér’s V = 0.134). However, regarding unstructured PA, boys significantly outperformed girls, *χ*^2^ (1, *N* = 138) = 4.08, *p* < 0.05 (Cramér’s V = 0.172).

#### 3.3.3. Weekly Duration of Physical Activity

The average time per week spent in structured and unstructured PA, respectively, are shown in Table 1. Children spent almost twice as much time engaging in unstructured PA compared to structured PA. The durations of structured and unstructured PA were uncorrelated (*r_s_* = −0.01, *p* = 0.89), emphasizing the limitation in leisure time disposable for engaging in PA.

A series of Mann-Whitney-U-Tests showed that boys spent significantly more time in structured (Z = −3.41, *p* < 0.001) as well as in unstructured PA (Z = −2.58, *p* < 0.05) compared to girls.

### 3.4. Structured vs. Unstructured Physical Activity and Mathematical Outcomes

Correlational analyses showed mathematical achievement to be positively correlated with time spent in structured PA (*r* = 0.17, *p* < 0.05), but uncorrelated with time spent in unstructured PA (*r* = 0.04, *p* = 0.62; *r* = 0.06, *p* = 0.48 after controlling for SES). Similarly, mathematical self-concept was only correlated with time spent in structured PA (*r_s_* = 0.19, *p* < 0.05), but uncorrelated with time spent in unstructured PA (*r_s_* = 0.12, *p* = 0.15; *r* = 0.12, *p* = 0.17 after controlling for SES). That is, although children spent almost twice as much time engaging in unstructured than in structured PA, only structured PA was significantly related to the measures of mathematical achievement and mathematical self-concept. For deeper insights regarding the relation between structured PA and mathematical outcomes, more detailed analyses seemed warranted.

### 3.5. Duration of Structured Physical Activity

#### 3.5.1. Structured Physical Activity and Mathematical Achievement

Comparing children who engaged in structured PA (i.e., children who were in at least one sports club; *M* = 24.78, *SD* = 4.67) with those who did not (*M* = 24.20, *SD* = 5.02), did not yield any difference regarding their mathematical achievement, *t*(136) = −0.62, *p* = 0.54, a finding that remained consistent even when considering girls and boys separately. However, among those children who were in a sports club, the time spent in structured PA was a significant factor in accounting for mathematical achievement. That is, the high-duration children (*M* = 25.62, *SD* = 4.57) achieved significantly higher scores on the mathematical achievement test than the low-duration children (*M* = 23.27, *SD* = 4.52), *t*(101) = −2.51, *p* < 0.05. In contrast, the low-duration children did not significantly differ from children who did not engage in structured PA at all, *t*(70) = 0.83, *p* = 0.41.

Rerunning the analyses for both genders separately showed the high-duration girls (*M* = 25.47, *SD* = 5.01) achieving significantly higher scores on the mathematical achievement test than the low-duration girls (*M* = 22.68, *SD* = 3.85), *t*(44) = −2.13, *p* < 0.05. In contrast, the high-duration boys (*M* = 25.70, *SD* = 4.40) did not differ significantly from the low-duration boys (*M* = 24.36, *SD* = 5.55) in terms of their mathematical achievement, *t*(55) = −0.91, *p* = 0.37.

#### 3.5.2. Structured Physical Activity and Mathematical Self-Concept

A Wilcoxon-Signed-Rank-Test showed that children who engaged in structured PA (i.e., children who were in at least one sports club; *M* = 3.74, *SD* = 1.18) and children who did not (*M* = 3.54, *SD* = 1.25), did not significantly differ in their mathematical self-concept, Z = −0.77, *p* = 0.44. With respect to time spent in structured PA, however, similar group differences were found for mathematical self-concept as with mathematical achievement. That is, the high-duration children (*M* = 4.02, *SD* = 1.06) had significantly higher mathematical self-concepts than the low-duration children (*M* = 3.24, *SD* = 1.23), Z = −3.35, *p* < 0.001. In contrast, the mathematical self-concept of the low-duration children did not differ from the mathematical self-concept of children who did not engage in structured PA at all, Z = −1.83, *p* = 0.07.

Again, the analyses were rerun for both genders separately. While the high-duration girls (*M* = 3.51, *SD* = 1.20) did not differ from the low-duration girls (*M* = 3.05, *SD* = 1.31) in their mathematical self-concept, Z = −1.22, *p* = 0.22, the high-duration boys (*M* = 4.27, *SD* = 0.90) had significantly higher mathematical self-concepts than the low-duration boys (*M* = 3.60, *SD* = 1.01), Z = −2.54, *p* < 0.05.

### 3.6. Self-Concept as a Mediator

**Figure 1:**
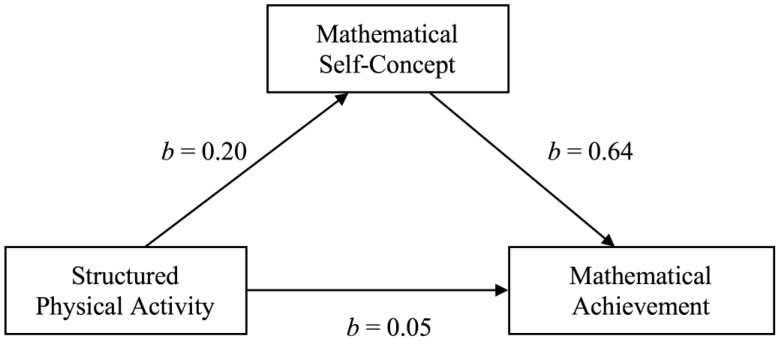
Mediational effect of time spent in structured physical activity (PA) on mathematical achievement by mathematical self-concept.

A Maximum Likelihood mediation analysis was used to investigate the hypothesis that mathematical self-concept mediated the effect of structured PA on mathematical achievement. A graphical illustration is provided in Figure 1. The results of a 2000 resampling bootstrapping procedure for the 95% bias-corrected confidence intervals indicated that time spent in structured PA was a significant predictor of mathematical self-concept (*b* = 0.20; CI = 0.077, 0.319) and that mathematical self-concept was a significant predictor of mathematical achievement (*b* = 0.64; CI = 0.530, 0.732). In the presence of the mediating effect, the direct effect of structured PA on mathematical achievement remained insignificant (*b* = 0.05; CI = −0.083, 0.181). The indirect, mediated effect of structured PA on mathematical achievement, however, was significant (CI = 0.052, 0.209), indicating a full positive mediation of structured PA on mathematical achievement by mathematical self-concept. 

A post-hoc power analysis using PASS 19.0.1 (NCSS, LLC, Kaysville, UT) revealed 82% power to detect a mediation effect of the reported size with a sample size of *N* = 138 with a two-sided alpha of 0.05.

Analyses of gender invariance showed the aforementioned mediation to be considered invariant for girls and boys, respectively, at the configural level, *χ*^2^_diff_ (52) = 49.60, *p* = 0.57. Moreover, metric invariance showed the construct relations (regression weights) to be at par for girls and boys, *χ*^2^_diff_ (12) = 10.00, *p* = 0.62. Thus, the reported mediation of structured PA on mathematical achievement by mathematical self-concept was equally applicable to boys and girls, even though the genders differed in the amount of weekly structured PA and the level of mathematical self-concept.

Although not significantly, the regression weights were slightly different for girls and boys, being higher for the boys than for the girls concerning time spent in structured PA on mathematical self-concept, and higher for the girls than for the boys regarding mathematical self-concept on mathematical achievement.

## 4. Discussion

The present study investigated the relationship between structured and unstructured PA and mathematical achievement in fourth-grade children. A growing body of research is hypothesizing a positive link between PA and academic outcomes; however, the findings are inconsistent, and the evidence for a general and direct relation between the constructs is less than clear [7,25]. Moreover, it remains to be explained how PA should be characterized—concerning its particular attributes like type and duration—to affect academic achievement [16]. In addressing these open issues, the present study sought to evaluate the effects of different types and durations of PA, as well as the indirect effect of PA on mathematical achievement as mediated by self-concept.

### 4.1. Structured and Unstructured Physical Activity

Regarding the type of PA, the results of the present study indicate that especially structured PA has the potential to contribute to mathematical performance. Although children spent almost twice as much time engaging in unstructured PA as compared to structured PA, only structured PA was significantly related to mathematical achievement. Similarly, only structured PA was related to mathematical self-concept—an important predictor for mathematical achievement as well. Thus, structured PA seems to feature characteristics that are essential for PA when it comes to its capacities to foster academic outcomes.

In line with previous research, these findings support the assumption of structured, hence adult-led, PA to be beneficial for academic outcomes [8]. Prior studies have shown that engaging in teacher- or trainer-guided PA may foster academic achievement. However, the majority of these studies have merely investigated school-based PA, such as physical education or extracurricular physical activities, provided by children’s and adolescent’s schools [7,8,11]. However, due to the high proximity to the educational environment, (structured) school-based PA has also been assumed to strengthen children’s sense of belonging to their school, thereby enhancing the motivation to succeed [6,21]. Thus, by relying on previous research, one cannot infer whether enhanced academic achievement is caused by PA per se or rather due to the schooling environment in which the activity is performed. As the present study was set out in Switzerland—a country where leisure PA takes place in school-independent settings—the reported relations between structured PA and mathematical achievement and mathematical self-concept, respectively, were unbiased by such indirect school effects. Hence, based on the present findings, it can be concluded that structured PA may have beneficial effects on academic outcomes, even when performed beyond and outside of school.

The finding that structured PA is related to mathematical achievement and self-concept, whereas unstructured PA is not, as well as the conclusion that the advantage of structured PA cannot be attributed to proximity to school, suggest that it is the structured PA that features essential characteristics when it comes to improving children’s academic outcomes. Based on the present study’s findings, such characteristics are assumed to lie in the organized and planned nature of structured PA; it takes place regularly, under guidance and control of an adult sports authority, and it is designed to improve one’s sport-specific skills [18,19]. Thus, while unstructured PA is mainly performed for having fun, engaging in structured PA includes the experience of consistently working toward progression and provides opportunities to experience what it means to pursue and achieve goals. Given these findings, we suppose that when regularly engaging in leisure activities aiming for self-improvement, children learn that they can succeed and achieve higher skills levels by investing time and effort. Such catalyzing effects emerging from PA may also transfer to the academic domain, improving children’s self-concept and motivation to learn and perform well. 

### 4.2. Duration of Physical Activity

Considering the relation between structured PA and mathematical outcomes in more detail, we compared children with different levels of temporal PA engagement. Overall, the results showed that children who did engage in structured PA and children who did not, neither differed in their mathematical self-concept nor their mathematical achievement. For the children who did engage in structured PA, however, the amount of time spent in structured PA constituted a critical factor in accounting for mathematical outcomes. That is, within a structured PA setting, the high-duration children had significantly higher mathematical self-concepts and achieved significantly higher scores on the mathematical achievement test than the low-duration children. Concerning the optimal duration of PA to empower children’s academic outcomes, we thus conclude that a setting of two hours or more weekly is needed to yield significant improvements.

In contrast to the hypothesis according to which high duration of PA might distract children from learning, the present findings indicate that it is virtually the high duration of PA that holds the potential to yield an enhancing effect on academic achievement at all. It has also been suggested that increasing PA shall be associated with improvements in academic achievement, but only so to an upper, however unknown, limit. Hence, exceeding an optimal level of PA is considered to no longer provide any additional benefits [16,24]. Although the existence of such a ceiling effect cannot be explicitly discarded given the present study, our findings contradict with the ceiling effect assumption, inasmuch as the children with the highest PA duration were shown to be both the ones with the highest mathematical self-concept as well as those attaining the highest scores on the mathematical achievement test.

### 4.3. Self-Concept as a Mediating Variable

As to the mediation by self-concept, results supported the claim of time spent in structured PA being indirectly related to mathematical achievement [26]. There was a full mediation of structured PA on mathematical achievement by mathematical self-concept. This finding held for both boys and girls. As the direct effect—i.e., structured PA on mathematical achievement—turned out to be insignificant when taking into account the mediation via mathematical self-concept, the self-concept enhancement effect due to PA seems to play a critical role in explaining the relation between structured PA and academic achievement.

The present study is one of the first of its kind, i.e., in explicitly investigating the mediational effect of self-concept given the relationship of PA and academic achievement [27]. Nonetheless, our results are in line with previous studies that, yet without having taken into account a mediational effect, reported positive effects of PA on self-concept [2,31]. Moreover, there is corroboration from previous research regarding the positive relationship between self-concept and academic achievement [32,33]. In search of the long-assumed mediation of PA on academic achievement by self-concept, the present study is finally extending and integrating on these independently accrued findings.

A further novel aspect addressed by the present study concerns the cross-domain relations of PA and self-concept. The majority of research in this area of interest has focused on the effects of PA on the physical self-concept. In line with Marsh [27], however, our findings support the assumption that PA has the potential not only to affect self-concept within the physical domain but also within the academic domain. In line with this claim, we found a small to moderate correlation between time spent in structured PA and mathematical self-concept, and self-concept, in turn, was strongly related to mathematical achievement. As discussed above, this finding indicates that regularly engaging in structured PA may help in empowering children’s confidence and motivation to invest time and effort to succeed in various domains. 

From a developmental perspective, the finding of a full mediation by self-concept also reflects important characteristics of the investigated age group. While physical health and cardiovascular fitness status—mechanisms known to be essential for the cognitive functioning and performance of adults and the elderly—are expected to be good for most children anyway [25], self-concept is known to decline during late childhood and adolescence [56]. Since self-concept is known to be substantially linked to academic achievement, the enhancement of self-concept through systematically organized and structured PA may constitute a crucial tool when it comes to supporting children along their school careers [24].

Although not the focus of the current study, we additionally explored the rationale behind the finding that PA affects mathematical achievement via mathematical self-concept. Therefore, explorative analyses were run to test Sonstroem’s assumption of a cascade starting with PA enhancing the physical self-concept that spills over into the general self-concept [28]. This, in turn, should lead to an enhanced mathematical self-concept, which is finally related to mathematical achievement. The results (see Appendix A) seem to confirm such a cascade within the assumption of a multidimensional self-concept [34].

### 4.4. Gender Differences

Overall, the reported findings were largely similar for boys and girls. Although boys had significantly higher mathematical self-concepts compared to girls, girls scored equally high on the mathematical achievement test. Although boys spent more time in both structured and unstructured PA than girls, only structured PA was related to mathematical self-concept and mathematical achievement with both genders. 

A gender difference concerning the amount of time needed to affect academic outcomes was revealed. As to the mathematical achievement, it was with the girls only that the high-duration group outperformed the low-duration group significantly. Regarding the mathematical self-concept, the difference between the aforementioned time categories was significant only with the boys. Thus, while the effect of structured PA on achievement was supposedly stronger for girls, the effect on self-concept was higher for boys. Regarding the partial lack of significance within these subsample analyses, it has to be stressed, however, that the small subsample sizes—with the low-duration boys’ group size of *n* = 13 being the smallest—cautioned to interpret the outcome of the analyses. Hence, the partial absence of substantial differences within subsamples may be due to insufficient power.

Although the above-noted finding that structured PA has a stronger effect on mathematical achievement in girls, yet a stronger effect on mathematical self-concept in boys, should be interpreted with caution, there are plausible reasons that may explain this difference. 

For one, there is gender-specific socialization that may contribute to the findings. On the one hand, engaging in PA still seems to constitute a masculine attribute [40,57], a fact that may explain why a boys’ self-concept is more strongly affected by structured PA than a girl’s self-concept. On the other hand, girls still face the stereotype of doing worse than boys in mathematical tasks [38], potentially rendering them susceptible to a self-fulfilling belief that may negatively affect the girls’ efforts in improving their mathematical self-concept.

For another, girls tend to engage in different sports activities than boys. While boys predominantly engage in team sports, such as soccer, basketball, or floorball, girls instead prefer individualistic sports, such as gymnastics, figure skating, or dancing. Research has shown that cooperative sports have more positive effects on self-concept, whereas individualistic, competitive sports could even have adverse effects on self-concept [58]. Thus, the unveiled stronger effect of structured PA on self-concept for boys, as compared to girls, could also be attributed to the engagement in different kinds of sports. Future studies investigating the link between PA and academic achievement should thus always take gender and specific sports types into consideration.

### 4.5. Limitations and Implications

The cross-sectional design of the present study limits the conclusions to mere correlational ones. Although in previous studies the direction has usually been assumed to run from PA to academic performance [25], it is impossible to infer the direction of any associations based on the present study. Thus, it may also be possible either that children with higher levels of self-concept have more confidence to engage in PA, or that children with better mathematical skills need less time to learn, e.g., mathematics, leaving them with more leisure time they can spend in PA. Therefore, future studies are advised to apply longitudinal designs to investigate the causal effects of structured PA on self-concept and academic achievement.

Regarding the partial lack of significance within subsample analyses, it has to be stressed that the limited statistical power due to the subsample’s sizes—coupled with small effect sizes—may have played a role in limiting the significance of some of the statistical analyses. Indeed, the effect sizes revealed in the present study were smaller than expected, and thus, subtle differences might not have been detected. Studies intending to replicate the present findings should thus aim for well-balanced subsample sizes allowing to compare the various subgroups with a higher power.

Finally, two issues concerning the assessment of PA shall be discussed. For one, it should be kept in mind that PA was assessed via self-report. Children may have reported higher activity times than they performed [46], and the method of assessment may have influenced the findings regarding the relation between PA and mathematical achievement [59]. For another, the current study concentrated on two specific subtypes of sports-related leisure-time PA, whereas PA can refer to a very broad range of bodily movement, including everyday activities, such as household tasks. Such everyday activities may best be assessed by applying up-to-date methods of objective activity tracking. Thus, future studies may reveal insightful findings by relying on accelerometer derived PA measures.

## 5. Conclusions

In conclusion, the current study extends the existing literature by scrutinizing three major open issues within the research field investigating the relation between PA and academic achievement. As to the question of how PA should be implemented to optimally foster children’s academic success, we investigated the roles of different types of PA, the needed duration to improve academic achievement, as well as the mediating role of self-concept. In sum, the results indicate that children benefit most from PA if (a) it is structured, (b) it lasts for at least two hours per week, and (c) it has empowering effects on children’s self-concept. Thus, engaging in high amounts of structured PA constitutes an advantageous leisure activity for children, not only to enhance their fitness and physical health but also to increase their self-concept and, finally, foster their academic success.

## Figures and Tables

**Table 1:** Descriptive statistics.

Variables	Total		Girls		Boys	
	*M*	*(SD)*	*M*	*(SD)*	*M*	*(SD)*
Structured PA (HPW)	2.47	(2.34)	1.79	(1.96)	3.11	(2.49)
Unstructured PA (HPW)	4.91	(7.99)	3.30	(6.45)	6.44	(8.99)
Mathematical achievement	24.63	(4.75)	23.94	(4.92)	25.28	(4.52)
Mathematical self-concept	3.69	(1.20)	3.32	(1.22)	4.04	(1.07)

Note: Means (*M*) and standard deviations (*SD*) for total sample (*N* = 138), girls (*n* = 67), and boys (*n* = 71). HPW = hours per week.

**Figure A1:**
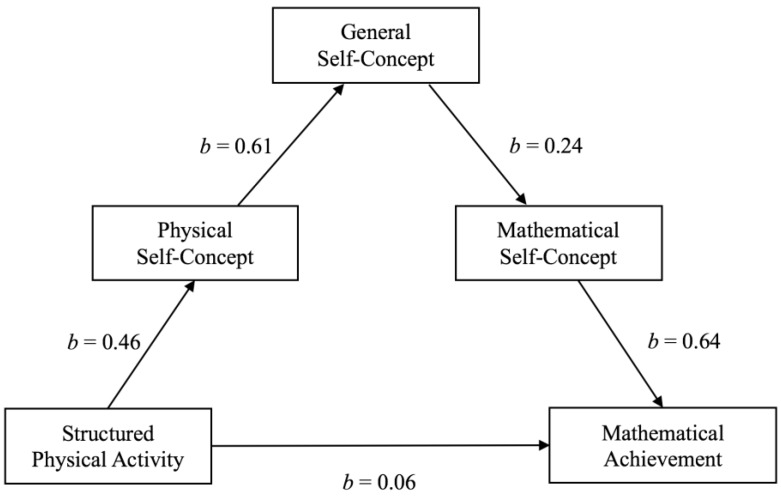
Evidence for the rationale behind the mediation by self-concept.

## Appendix A

Note: Additional exploratory analyses were run on the rationale behind the assumption that self-concept is mediating the relationship between PA and mathematical achievement. Theoretical assumptions are based on the exercise and self-esteem model [28] within a multidimensional self-concept [34]. Physical self-concept and general self-concept were measured by eight items of the respective subscale of the SDQ-I each [47,48]. A Maximum Likelihood serial mediation analysis with latent variables was applied in Amos software [53]. The results of a 2000 resampling bootstrapping procedure for the 95% bias-corrected confidence intervals indicated that time spent with structured PA was a significant predictor of physical self-concept (*b* = 0.46; CI = 0.001, 0.003), physical self-concept was a significant predictor of general self-concept (*b* = 0.61; CI = 0.297, 0.861), general self-concept was a significant predictor of mathematical self-concept (*b* = 0.24; CI = 0.102, 0.864), and mathematical self-concept was a significant predictor of mathematical achievement (*b* = 0.64; CI = 1.977, 3.173). In the presence of the serial mediating effect, the direct effect of structured PA on mathematical achievement remained insignificant (*b* = 0.06; CI = −0.071, 0.200). The indirect, mediated effect of structured PA on mathematical achievement, however, was significant (CI = 0.011, 0.095), indicating a full positive serial mediation of structured PA on mathematical achievement via the assumed path.
